# Circulating tumor cells are an indicator for the administration of adjuvant transarterial chemoembolization in hepatocellular carcinoma: A single‐center, retrospective, propensity‐matched study

**DOI:** 10.1002/ctm2.137

**Published:** 2020-07-23

**Authors:** Peng‐Xiang Wang, Yun‐Fan Sun, Kai‐Qian Zhou, Jian‐Wen Cheng, Bo Hu, Wei Guo, Yue Yin, Jun‐Feng Huang, Jian Zhou, Jia Fan, Tan To Cheung, Xu‐Dong Qu, Xin‐Rong Yang

**Affiliations:** ^1^ Department of Liver Surgery & Transplantation, Liver Cancer Institute, Zhongshan Hospital Fudan University Shanghai China; ^2^ Key Laboratory of Carcinogenesis and Cancer Invasion Ministry of Education Shanghai China; ^3^ Department of Laboratory Medicine, Zhongshan Hospital Fudan University Shanghai China; ^4^ Department of Intensive Care Medicine, Zhongshan Hospital Fudan University Shanghai China; ^5^ Institutes of Biomedical Sciences Fudan University Shanghai China; ^6^ Department of Surgery The University of Hong Kong Hong Kong China; ^7^ Department of Intervention Radiology, Zhongshan Hospital Fudan University Shanghai China

**Keywords:** a propensity score matching analysis, adjuvant transcatheter arterial chemoembolization, circulating tumor cells, hepatocellular carcinoma

## Abstract

**Background:**

High rates of postoperative tumor recurrence contribute to poor outcome in hepatocellular carcinoma (HCC). Here, we investigated whether circulating tumor cells (CTCs) status can predict the benefit of adjuvant transcatheter arterial chemoembolization (TACE) in patients with HCC.

**Methods:**

The retrospective study enrolled 344 HCC patients with preoperative CTCs analysis. Clinical outcomes including recurrence and survival were compared between those who received and who did not receive adjuvant TACE. Similar comparisons were made for patients stratified according to CTC status (CTC‐negative [CTC = 0], n = 123; CTC‐positive [CTC ≥ 1], n = 221). Propensity score matching (PSM) strategy was adopted to offset differences between two groups.

**Results:**

In the study cohort as a whole or in CTC‐negative cohort, there were no observable differences in overall survival (OS) or time to recurrence (TTR) between TACE and control group (*P *> .05). In CTC‐positive patients, PSM generated 64 patient pairs, and patients with adjuvant TACE had significantly better clinical outcomes (OS: not reached vs 36.4 months, *P *< .001; TTR: 45.8 vs 9.8 months, *P *< .001). Adjuvant TACE significantly reduced early recurrence (≤2 years) (64.1% vs 31.7%, *P *< .001) in CTC‐positive patients. Notably, adjuvant TACE influenced TTR and OS even in subgroups of CTC‐positive patients with low risk of recurrence according to traditional evaluation.

**Conclusions:**

Preoperative CTC status could serve as an indicator for the administration of adjuvant TACE in HCC patients. Adjuvant TACE benefits CTC‐positive HCC patients mainly by reducing early recurrence.

## Graphical abstract

No uniform criteria had been established for the administration of adjuvant transcatheter arterial chemoembolization (TACE) for hepatocellular carcinoma (HCC) patients after surgery. Results from this large‐sample, retrospective study showed that adjuvant TACE can significantly reduce tumor recurrence for circulating tumor cell‐positive patients. TACE can reduce tumor early recurrence and improve clinical outcomes for selected HCC patients.

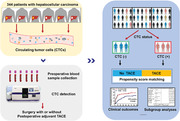

## 1 INTRODUCTION

Surgical resection remains the optimal treatment option for hepatocellular carcinoma (HCC).^1^ Nevertheless, the 5‐year cumulative recurrence rate is 50‐70% even after curative surgical resection, which resulted in the poor prognosis.^2^, ^3^ For approximately 70% of cases, tumor recurrence occurs within 2 years after surgery and is primarily due to postoperative minimal residual disease (MRD).^4^, ^5^ A previous retrospective study demonstrated that adjuvant transcatheter arterial chemoembolization (TACE) could prolong overall survival (OS) for patients who were likely to experience tumor recurrence after surgery.^6^ Moreover, our recent randomized prospective controlled study also confirmed that adjuvant TACE can significantly improve OS via reducing early tumor recurrence in hepatitis B virus‐associated HCC at moderate or high risk of recurrence postsurgery.^7^ Unfortunately, the current clinical use of adjuvant TACE primarily depends on the experience of the clinicians based on the assessment of traditional tumor characteristics, and it remains very difficult to predict whether a patient will benefit from postoperative adjuvant TACE treatment precisely.^8^ Therefore, there is an urgent need to identify reliable indicators to guide postoperative adjuvant TACE interventions for HCC.

Our previous study confirmed that the high circulating tumor cell (CTC) count was related to tumor recurrence after surgical resection in HCC patients.^9^ In the same study, we also noticed that adjuvant TACE exhibited a potential benefit in reducing recurrence in patients who had high preoperative CTC burden.^9^ Although the short follow‐up period and the cohort size of this study impeded a definitive conclusion from being made, the data showed that preoperative CTC status may be useful to indicate the benefit of postoperative adjuvant TACE for HCC patients.

Optimally, a prospective, randomized controlled study should be used to investigate whether CTC status is a clinical indicator for the benefit of adjuvant TACE, but such study proved to be great challenging.^10^ As an alternative, propensity score matching (PSM) can help to minimize patients selection bias and overcome the discrepancies of clinical characteristics among groups.^10^ In this study, we analyzed the data from 344 HCC patients with preoperative CTC analysis at our institute.

## 2 MATERIALS AND METHODS

### 2.1 Patient enrollment

**FIGURE 1:**
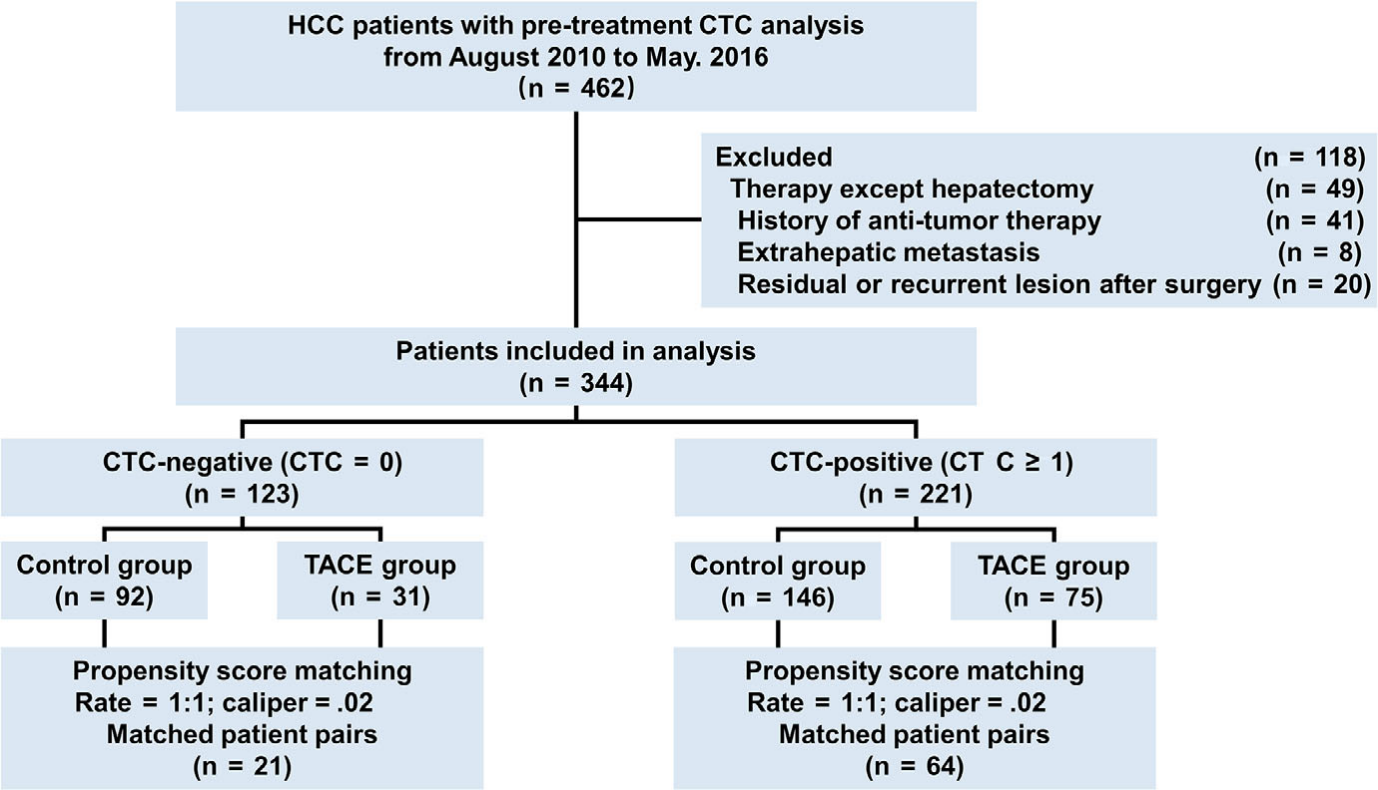
Flow chart for patient selection in this study

A total of 344 HCC patients with preoperative CTC analysis at our institute from August 2010 to May 2016 were retrospectively reviewed and considered for inclusion in this study. In all patients, HCC was confirmed by histological examination. The inclusion criteria were as follows: (a) no prior antitumor treatment; (b) liver function of Child‐Pugh class A or B; (c) HCC treated by curative‐intent partial hepatectomy, which was defined as resection of all macroscopic lesions (surgical margin was free of residual cancer cells based on histological examination); (d) no extrahepatic metastasis or other malignancies; (e) no residual cancer or HCC recurrence was diagnosed at first postoperative follow‐up at 1 month after surgery; and (f) no serious major organ dysfunctions. Barcelona Clinic Liver Cancer (BCLC) stage^11^ and the Guidelines of Primary Liver Cancer in China (CNLC)^12^ were used to determine tumor stage. Edmondson grade was used to determine tumor differentiation. Liver function was evaluated using the Child‐Pugh score. Figure 1 showed the study design.

The study was approved by the Research Ethics Board of Zhongshan Hospital, with written informed consent obtained from every patient.

### 2.2 Follow‐up and tumor recurrence

Patients who needed antiviral therapy were given either entecavir (0.5‐1.0 mg) or lamivudine (100 mg) per day after surgery. Adefovir (10 mg) per day was added if the drug resistance occurred.^13^

Postoperative patient surveillance was conducted as described previously.^9^ The first postoperative follow‐up was conducted within first month (3‐4 week) after surgery; every patient received a comprehensive evaluation, including alpha‐fetoprotein (AFP) test, postoperative liver function, and abdominal ultrasonography. If any residual lesion or recurrence was suspected, enhanced magnetic resonance imaging (MRI) or computed tomography (CT) would then be performed. Patients who had residual lesion or recurrence according to the image examinations would be excluded from the study. Further follow‐ups occurred every 2‐3 months in the first year postsurgery and every 3‐4 months for the following years, with follow‐ups for this study ending in August 2019. Each routine follow‐up visit included the following evaluations: measurement of serum AFP level, liver function test, abdominal ultrasonography, and chest radiography. If recurrence was suspected, abdominal CT, MRI, or a bone scan would be performed immediately. In addition, biopsy would be performed if necessary.

HCC recurrence was diagnosed based on the China Guidelines for Diagnosis and Treatment of Primary Liver Cancer in China (2017 Edition).^12^ Treatment strategies for tumor recurrence were chosen case‐by‐case. Patients were offered additional liver resection, radiofrequency ablation, TACE, radiotherapy, or sorafenib depending on lesion number, lesion diameter, and liver function status.

OS was the interval from the date of surgery to either the date of death or the last follow‐up visit. Time to recurrence (TTR) was the time between resection and the occurrence of any type of recurrence, and a 2‐year threshold was used as discriminative cut‐off value for early and late recurrence.^14^

### 2.3 Adjuvant TACE procedures

Risk of HCC recurrence was evaluated by physicians primarily based on tumor features according to the pathology report. Patients with high recurrence risk (with one or multiple following features: tumor diameter ≥ 5 cm, multiple tumors, incomplete tumor capsule, Edmondson stage III‐IV, tumor with vascular invasion, or advanced tumor stage) were appropriately recommended to receive an adjuvant TACE after informing them of the potential risks and benefits.^1^, ^12^

After the first postoperative follow‐up evaluation, one course of adjuvant TACE was conducted for those with no residual lesion or recurrence in the entire liver remnant within 4‐6 weeks after surgery using the Seldinger technique.^7^, ^15^, ^16^ Briefly, a catheter was introduced through the femoral artery. Superior mesenteric artery angiography or hepatic angiography was conducted to show any tumor stains in the liver. Hepatic arterial catheterized infusion of chemotherapy for whole liver was performed through proper hepatic artery for all patients with 5‐fluorouracil (500 mg/m^2^), or oxaliplatin (100 mg/m^2^) first. The regimen of hepatic arterial catheterized infusion was adjusted according to the liver function, peripheral leukocyte, and platelet counts of the patients. Then, preventive or therapeutic lipiodol chemoembolization was performed based on whether a suspected target tumor stain was detected in the liver remnant.^15^ Preventive lip‐chemoembolization was performed if no obvious tumor stain was observed; selective catheterization through left and right hepatic arteries was used to inject an emulsion consisting of lipiodol (3‐5 mL) and epirubicin (30 mg/m^2^) to both lobes. The volume of lipiodol injected was adjusted according to the volume of the remnant liver. Therapeutic chemoembolization was performed by administrating super‐selective arterial catheterized infusion of the emulsion consisting of epirubicin and lipiodol via tumor artery or regional supplying artery. The dosage was individually adjusted based on the tumor volume and liver function of patients.

### 2.4 CTC isolation and detection

CTC analysis was performed 2 days preoperatively using the CellSearch system (Menarini Silicon Biosystems).^9^ Briefly, 7.5 mL blood was collected in CellSave (Menarini Silicon Biosystems) tubes and processed according to the standard manufacturer's guidance. CTCs were isolated, immunofluorescent‐stained with antibodies against epithelial cell adhesion molecule (EpCAM), cytokeratin 8, 18, 19, CD45, and 4,6‐diamidino‐2‐phenylindole (DAPI), and then enumerated by semi‐automated four‐color fluorescence microscopy. CTC is defined as an intact nucleated cell (DAPI^+^), positive for cytokeratin 8/18/19 and/or EpCAM, and negative for the leukocyte‐biomarker CD45. Patient with CTC ≥ 1 was considered as CTC‐positive.

### 2.5 PSM analysis

Because adjuvant TACE treatment was not randomly assigned to HCC patients, PSM was conducted to minimize patient selection bias and eliminate potential confounders.^10^ Logistic regression model was adopted to calculate the propensity scores (from 0 to 1) and patients in TACE group were paired with those without TACE at 1:1 ratio. Nearest‐neighbor matching algorithm was adopted (caliper width = 0.02). Variables that may influence the decision of adjuvant TACE and clinical outcomes based on clinical knowledge were included into the PSM model, that included the following variables: gender (male or female), age (≤50 or >50 years), tumor number (single or multiple), tumor diameter (≤5 or >5 cm), tumor encapsulation (none or complete), vascular invasion (no or yes), Edmondson stage (I‐II or III‐IV), liver cirrhosis (no or yes), hepatitis B virus infection (no or yes), AFP level (≤400 or >400 ng/mL), alanine aminotransferase (ALT) level (≤50 or >50 U/L), gamma‐glutamyl transpeptidase (GGT) level (≤60 or >60 U/L), and Child‐Pugh class (A or B).

### 2.6 Statistical analysis

Values were presented as mean ± standard deviations. Variables were compared using the Student's *t* test, Wilcoxon signed‐rank test, *χ* ^2^ test, or Fisher's exact test. The survival curves were estimated by Kaplan‐Meier analysis and compared using log‐rank test. Cox regression model was used in univariate analyses for risk factors. Subgroup analyses were conducted in patients stratified by clinical clinicopathological factors, and treatment efficacy was evaluated using multiple individual Cox models. Statistical analysis was conducted using SPSS 24.0 (IBM). *P*‐value (two‐tailed) < .05 was considered statistically significant.

## 3 RESULTS

### 3.1 Baseline characteristics of patients with HCC

The baseline clinical characteristics of the HCC patients for the entire cohort are presented in Table 1. Clinical features of the patients stratified by different preoperative CTC status are presented in Tables 2 and S1. Of the 344 HCC patients enrolled in the study, 106 patients (30.8%) received postoperative adjuvant TACE. The median follow‐up time was 45.0 months for the TACE group and 48.9 months for the control group. A total of 114 patients died in the follow‐up, including 82 patients (34.5%) in the control group and 32 patients (30.2%) in the TACE group, and 106 of these 114 (93.0%) patients had suffered HCC progression during the study period. Moreover, a total of 187 patients (54.4%) had tumor recurrence at the end of follow‐up, including 60 of 106 (56.6%) patients in the TACE group and 127 of 238 (53.4%) patients in the control group. Most of the recurrences were intrahepatic recurrence alone (70.1% for control group and 66.7% for TACE group; Table 3). Kaplan‐Meier analysis revealed that adjuvant TACE did not show therapeutic benefit for OS or TTR in the study cohort as a whole (*P *> .05; Figure S1A and S1B).

**TABLE 1:** Baseline characteristics of HCC patients for the entire cohort

		n = 344	
Variable		n	%
Gender	Male	296	86.0%
	Female	48	14.0%
Age (years)	≤50	141	41.0%
	>50	203	59.0%
CTC Count	<1	123	35.8%
	≥1	221	64.2%
Tumor number	Single	259	75.3%
	Multiple	85	24.7%
Tumor diameter (cm)	≤5	219	63.7%
	>5	125	36.3%
Tumor capsule	Complete	206	59.9%
	None	138	40.1%
Vascular invasion	No	203	59.0%
	Yes	141	41.0%
Edmondson stage	I‐II	209	60.8%
	III‐IV	135	39.2%
Liver cirrhosis	No	154	44.8%
	Yes	190	55.2%
HBsAg	Negative	49	14.2%
	Positive	295	85.8%
AFP (ng/mL)	≤400	240	69.8%
	>400	104	30.2%
ALT (U/L)	≤50	274	79.7%
	>50	70	20.3%
GGT (U/L)	≤60	202	58.7%
	>60	142	41.3%
Child‐Pugh class	A	339	98.5%
	B	5	1.5%
BCLC stage	0‐A	254	73.8%
	B‐C	90	26.2%
CNLC stage	I	254	73.8%
	II‐III	90	26.2%
TACE	No	238	69.2%
	Yes	106	30.8%

Abbreviations: AFP, alpha‐fetoprotein; ALT, alanine aminotransferase; BCLC, Barcelona Clinic Liver Cancer staging system; CNLC, Liver Cancer Guidelines in China; CTC, circulating tumor cell; GGT, gamma‐glutamyl transpeptidase; HBsAg, Hepatitis B surface antigen; HCC, hepatocellular carcinoma; TACE, transcatheter arterial chemoembolization.

**TABLE 2:** Baseline characteristics in CTC‐positive HCC patients

		Before propensity matching (n = 221)			After propensity matching (n = 128)		
		Control	TACE		Control	TACE	
Variable		(n = 146)	(n = 75)	*P*‐value	(n = 64)	(n = 64)	*P*‐value
Gender	Male	124 (84.9%)	62 (82.7%)	.662	54 (84.4%)	55 (85.9%)	.804
	Female	22 (15.1%)	13 (17.3%)		10 (15.6%)	9 (14.1%)	
Age (years)	≤50	63 (43.2%)	32 (42.7%)	.945	27 (42.2%)	26 (40.6%)	.858
	>50	83 (56.8%)	43 (57.3%)		37 (57.8%)	38 (59.4%)	
CTC count	Mean ± SD	4.17 ± 7.47	4.89 ± 9.25	.554	5.03 ± 10.07	5.42 ± 9.91	.931
Tumor number	Single	112 (76.7%)	47 (62.7%)	**.028**	39 (60.9%)	40 (62.5%)	.856
	Multiple	34 (23.3%)	28 (37.3%)		25 (39.1%)	24 (37.5%)	
Tumor diameter (cm)	≤5	91 (62.3%)	37 (49.3%)	.064	32 (50.0%)	31 (48.4%)	.860
	>5	55 (37.7%)	38 (50.7%)		32 (50.0%)	33 (51.6%)	
Tumor capsule	Complete	93 (63.7%)	45 (60.0%)	.591	40 (62.5%)	41 (64.1%)	.855
	None	53 (36.3%)	30 (40.0%)		24 (37.5%)	23 (35.9%)	
Vascular invasion	No	94 (64.4%)	27 (36.0%)	**<.001**	23 (35.9%)	26 (40.6%)	.585
	Yes	52 (35.6%)	48 (64.0%)		41 (64.1%)	38 (59.4%)	
Edmondson stage	I‐II	93 (63.7%)	39 (52.0%)	.093	38 (59.4%)	34 (53.1%)	.476
	III‐IV	53 (36.3%)	36 (48.0%)		26 (40.6%)	30 (46.9%)	
Liver cirrhosis	No	60 (41.1%)	35 (46.7%)	.428	26 (40.6%)	29 (45.3%)	.592
	Yes	86 (58.9%)	40 (53.3%)		38 (59.4%)	35 (54.7%)	
HBsAg	Negative	19 (13.0%)	10 (13.3%)	.947	8 (12.5%)	9 (14.1%)	.795
	Positive	127 (87.0%)	65 (86.7%)		56 (87.5%)	55(85.9%)	
AFP (ng/mL)	≤400	97 (66.4%)	51 (68.0%)	.815	36 (56.3%)	43 (67.2%)	.203
	>400	49 (33.6%)	24 (32.0%)		28 (43.7%)	21 (32.8%)	
ALT (U/L)	≤50	112 (76.7%)	59 (78.7%)	.742	46 (71.9%)	50 (78.1%)	.414
	>50	34 (23.3%)	16 (21.3%)		18 (28.1%)	14 (21.9%)	
GGT (U/L)	≤60	83 (56.8%)	46 (61.3%)	.522	36 (56.3%)	38 (59.4%)	.720
	>60	63 (43.2%)	29 (38.7%)		28 (43.7%)	26 (40.6%)	
Child‐Pugh class	A	144 (98.6%)	74 (98.8%)	1.000^a^	63 (98.4%)	63 (98.4%)	1.000^a^
	B	2 (1.4%)	1 (1.2%)		1 (1.6%)	1 (1.6%)	
BCLC stage	0‐A	109 (74.7%)	44 (58.7%)	**.015**	38 (59.4%)	38 (59.4%)	1.000
	B‐C	37 (25.3%)	31 (41.3%)		26 (40.6%)	26 (40.6%)	
CNLC stage	I	109 (74.7%)	44 (58.7%)	**.015**	38 (59.4%)	38 (59.4%)	1.000
	II‐III	37 (25.3%)	31 (41.3%)		26 (40.6%)	26 (40.6%)	

a Continuous correction.

Bold *P*‐values indicates statistical significance.

Abbreviations: AFP, alpha‐fetoprotein; ALT, alanine aminotransferase; BCLC, Barcelona Clinic Liver Cancer staging system; CNLC, Liver Cancer Guidelines in China; CTC, circulating tumor cell; GGT, gamma‐glutamyl transpeptidase; HBsAg, Hepatitis B surface antigen; HCC, hepatocellular carcinoma; TACE, transcatheter arterial chemoembolization.

**TABLE 3:** Location of recurrent hepatocellular carcinoma

	Control	TACE	
Variable	(n = 238, %)	(n = 106, %)	*P*‐value
No. of recurrence	127	60	
Location of recurrence			.808^a^
Intrahepatic	89 (70.1%)	40 (66.7%)	
Extrahepatic	5 (3.9%)	3 (5.0%)	
Both intrahepatic and extrahepatic	33 (26.0%)	17 (28.3%)	

a Fisher's exact test.

Abbreviation: TACE, transcatheter arterial chemoembolization.

To determine whether CTC status could identify patients that benefited from adjuvant TACE, the study cohort was stratified according to patient CTC status. Of the 123 patients who were CTC‐negative (CTC = 0), 31 patients had adjuvant TACE and 92 patients did not. Of the 221 patients who were CTC‐positive (CTC ≥ 1), 75 patients received postoperative adjuvant TACE and 146 patients did not. Additionally, patients receiving adjuvant TACE treatment tended to have more advanced HCC (Tables 2 and S1).

### 3.2 Pre‐PSM assessment of the clinical treatment efficacy of postoperative TACE

**FIGURE 2:**
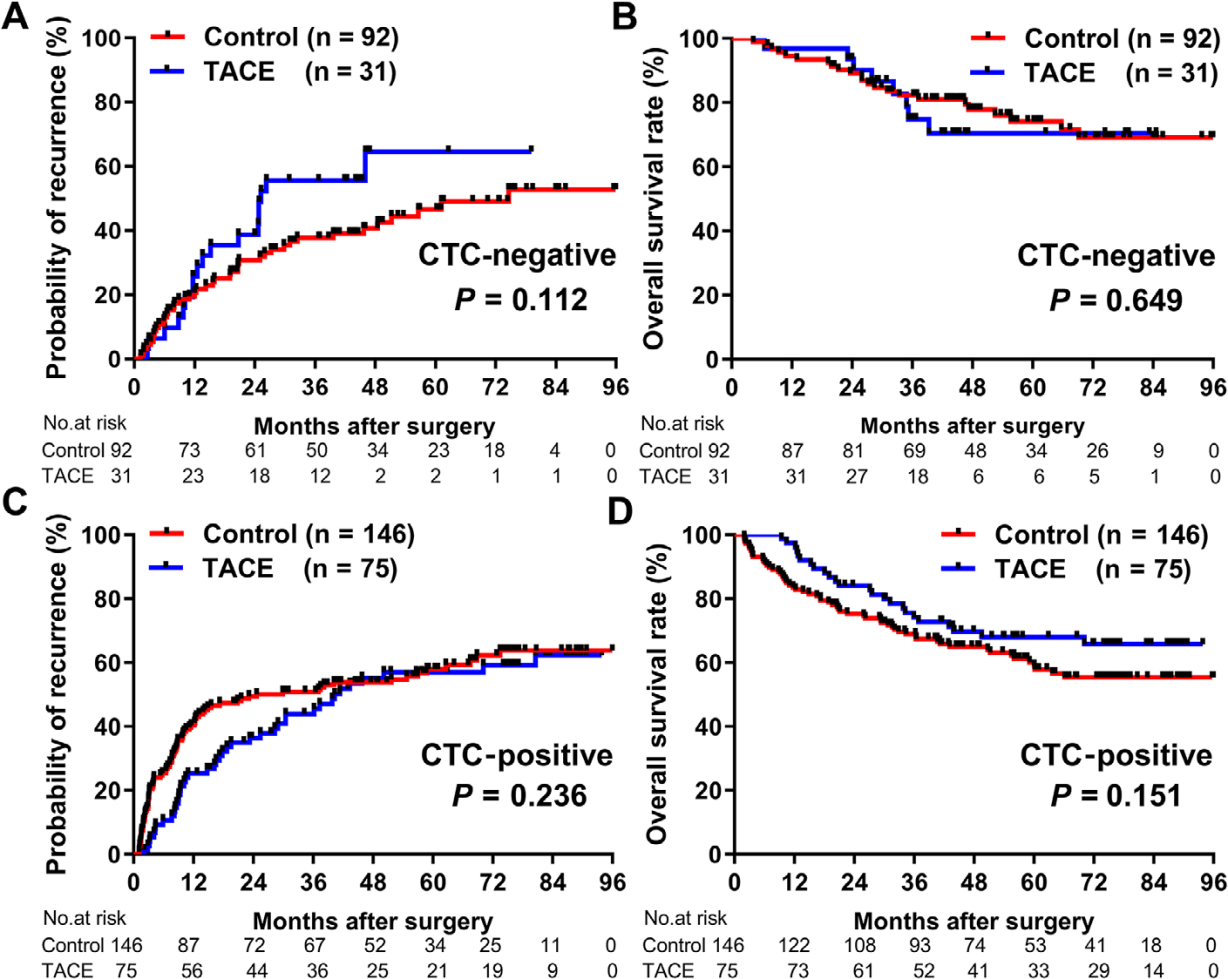
Comparison of recurrence rates and OS between CTC‐stratified HCC patients in TACE and control groups before PSM. Recurrence rates (A) and OS (B) for CTC‐negative patients in TACE and control groups before PSM. Recurrence rates (C) and OS (D) for CTC‐positive patients in TACE and control groups before PSM

For CTC‐negative patients, the recurrence rate (64.6% vs 52.9%, *P *= .112) or median OS (both not reached, *P *= .649) was not significantly different between the TACE and control groups (Figures 2A and 2B). Similarly, for CTC‐positive patients, the median TTR and recurrence rate were not significantly different between groups (median TTR: 40.3 vs 24.7 months, *P *= .236; Figure 2C). Additionally, for median OS, no significant difference between the patients with TACE treatment and those without was observed (both not reached, *P *= .151; Figure 2D).

### 3.3 Post‐PSM assessment of the treatment efficacy of postoperative TACE

Using PSM, we generated 64 matched pairs of CTC‐positive patients and 21 matched pairs of CTC‐negative patients. There was no significant difference for any clinical characteristics between the TACE and control groups (Tables 2 and S1).

**FIGURE 3:**
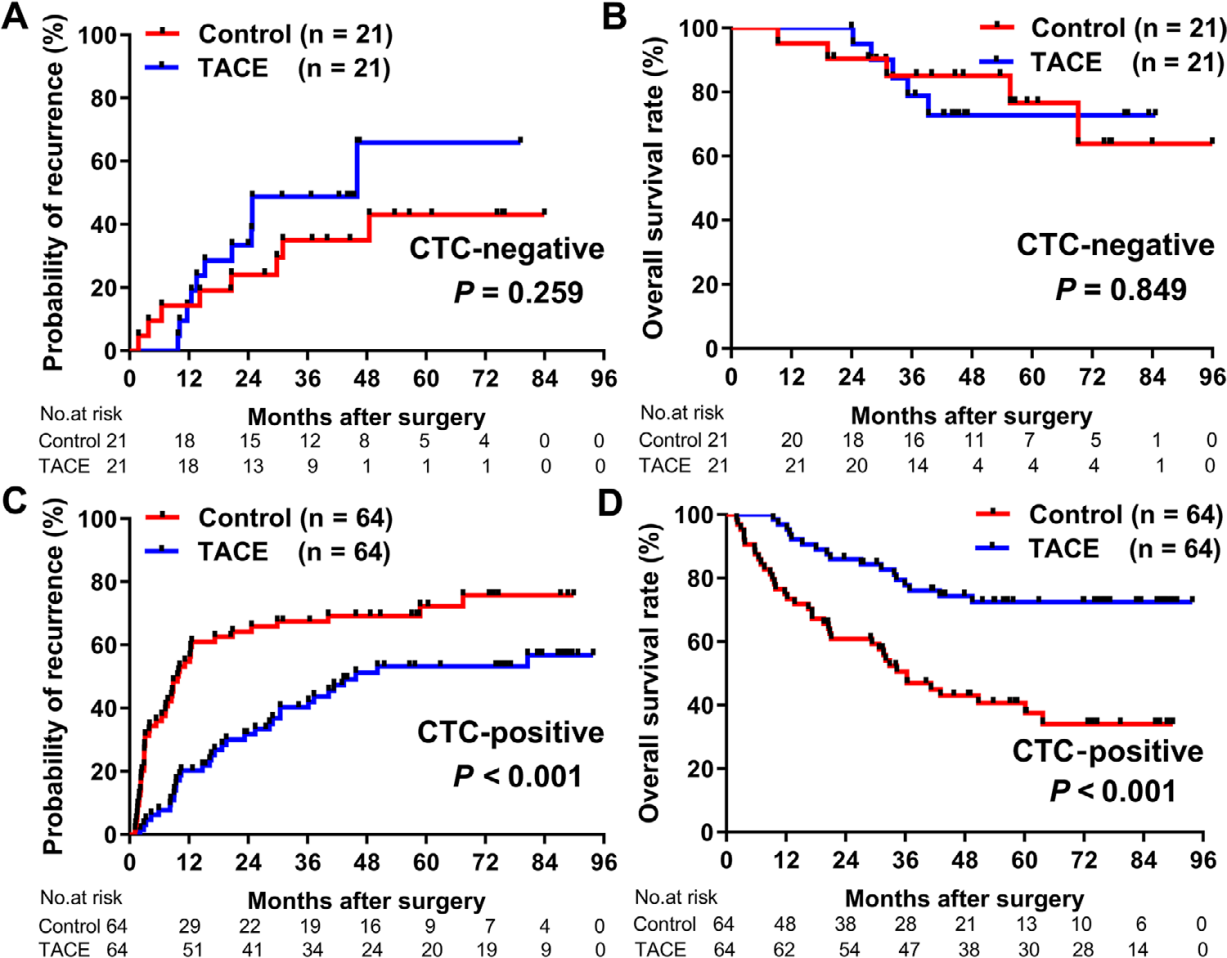
Comparison of recurrence rates and OS between CTC‐stratified HCC patients in TACE and control groups after PSM. Recurrence rates (A) and OS (B) for CTC‐negative patients in TACE and control groups after PSM. Recurrence rates (C) and OS (D) for CTC‐positive patients in TACE and control groups after PSM

For CTC‐negative patients, analysis of the matched cohort yielded similar results to those of pre‐PSM analysis; no significant difference between the TACE group and control group for either median TTR (46.3 months vs not reached, *P *= .259; Figure 3A) or median OS was observed (both not reached, *P *= .849; Figure 3B).

For the matched CTC‐positive patients, the recurrence rates at 1 year, 3 years, and 5 years differed between the TACE group (20.3%, 40.2%, and 53.2%, respectively) and the control group (54.7%, 67.4%, and 72.3%, respectively; Figure 3C). Additionally, CTC‐positive patients who had adjuvant TACE treatment had significantly longer median TTR than patients in the control group (45.8 vs 9.8 months, *P *< .001). Besides, with regard to OS, the 1‐year, 3‐year, and 5‐year survival rates in the TACE group were 96.9%, 77.7%, and 72.4%, respectively, and 75.0%, 50.5%, and 40.6% in the control group, respectively; the median OS of the patients with adjuvant TACE treatment (not reached) was significantly longer than those in the control group (36.4 months, *P *< .001; Figure 3D).

Univariate analysis was used to assess the effect of TACE on tumor recurrence and OS in CTC‐positive patients after PSM. We found that adjuvant TACE was correlated with a 53.9% risk reduction of tumor recurrence (hazard ratio [HR] = .461; 95% confidence interval [CI], .294‐.723; *P *= .001). Other covariates including tumor number, tumor diameter, liver cirrhosis, AFP level, ALT level, and GGT level were also associated with TTR (*P *< .05 for each covariate; Table 4). Additionally, univariate analysis also revealed an association between adjuvant TACE and OS (HR = .316; 95% CI, .178‐.562; *P *< .001; Table 4).

**TABLE 4:** Univariate analyses to identify independent risk factors of time to recurrence and overall survival in CTC‐positive HCC patients after PSM

	Time to recurrence		Overall survival	
Variable	HR (95% CI)	*P*‐value	HR (95% CI)	*P*‐value
Gender (Male)	1.142 (0.603‐2.161)	.684	0.822 (0.414‐1.631)	.575
Age (>50 years)	0.747 (0.479‐1.166)	.199	0.618 (0.364‐1.049)	.075
Tumor number (Multiple)	2.087 (1.336‐3.260)	**.001**	2.540 (1.491‐4.328)	**.001**
Tumor diameter (>5 cm)	1.604 (1.027‐2.504)	**.038**	2.057 (1.191‐3.553)	**.010**
Tumor capsule (None)	1.345 (0.858‐2.109)	.196	1.389 (0.812‐2.374)	.230
Vascular invasion (Yes)	1.312 (0.827‐2.082)	.248	1.241 (0.716‐2.150)	.442
Edmondson stage (III‐IV)	0.883 (0.562‐1.388)	.590	1.161 (0.683‐1.973)	.582
Liver cirrhosis (Yes)	1.702 (1.073‐2.701)	**.024**	1.517 (0.870‐2.645)	.142
HBsAg (Positive)	1.586 (0.763‐3.298)	.217	1.147 (0.491‐2.681)	.751
AFP (>400 ng/mL)	2.314 (1.481‐3.615)	**<.001**	3.352 (1.953‐5.753)	**<.001**
ALT (>50 U/L)	2.350 (1.473‐3.749)	**<.001**	2.330 (1.356‐4.003)	**.002**
GGT (>60 U/L)	1.687 (1.084‐2.625)	**.020**	1.894 (1.114‐3.219)	**.018**
Adjuvant TACE (Yes)	0.461 (0.294‐0.723)	**.001**	0.316 (0.178‐0.562)	**<.001**

Bold *P*‐values indicates statistical significance.

Abbreviations: AFP, alpha‐fetoprotein; ALT, alanine aminotransferase; BCLC, Barcelona Clinic Liver Cancer staging system; CNLC, Liver Cancer Guidelines in China; CTC, circulating tumor cell; GGT, gamma‐glutamyl transpeptidase; HBsAg, Hepatitis B surface antigen; HCC, hepatocellular carcinoma; TACE, transcatheter arterial chemoembolization.

### 3.4 Adjuvant TACE reduces early recurrence in CTC‐positive patients

**FIGURE 4:**
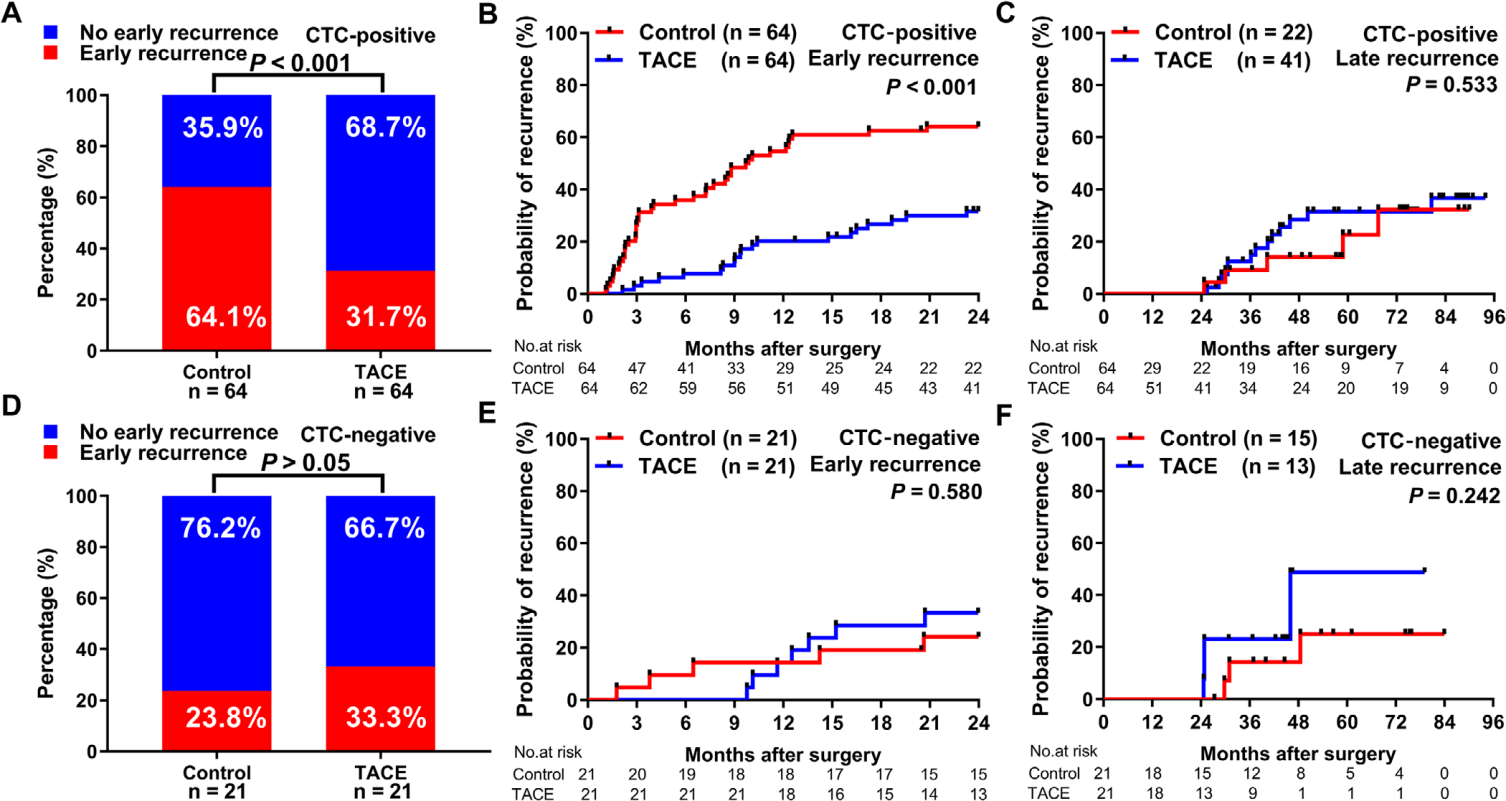
Associations between adjuvant TACE and early recurrence in CTC‐stratified HCC patients. A, Proportions CTC‐positive patients in TACE and control groups that experienced early recurrence or nonearly recurrence. Kaplan‐Meier analysis of rates of early recurrence (B) and late recurrence (C) for CTC‐positive patients in TACE and control groups. D, Proportions of CTC‐negative patients in TACE and control groups that experienced early recurrence or nonearly recurrence. Kaplan‐Meier analysis of rates of early recurrence (E) and late recurrence (F) for CTC‐negative patients in TACE and control groups

We next investigated the association between adjuvant TACE and postoperative tumor recurrence in HCC patients stratified by CTC status. Using 2 years as threshold, early recurrence was observed in 61 (47.7%) of the 128 matched CTC‐positive patients and in 12 (28.6%) of the 42 matched CTC‐negative patients. For the CTC‐positive patients, we found that the early recurrence (≤2 years) rates of the adjuvant TACE group (31.7%) were significantly lower than control group (64.1%, *P *< .001), whereas the late recurrence (>2 years) rates between the groups were similar (36.8% vs 32.4%; *P *= .533; Figure 4A‐C). However, considering the relative low recurrence rates in CTC‐negative patients, only seven (33.3%) of the 21 patients in the TACE group and five (23.8%) of the 21 patients in the control group experienced early recurrence (*P *= .580; Figure 4D‐F).

**FIGURE 5:**
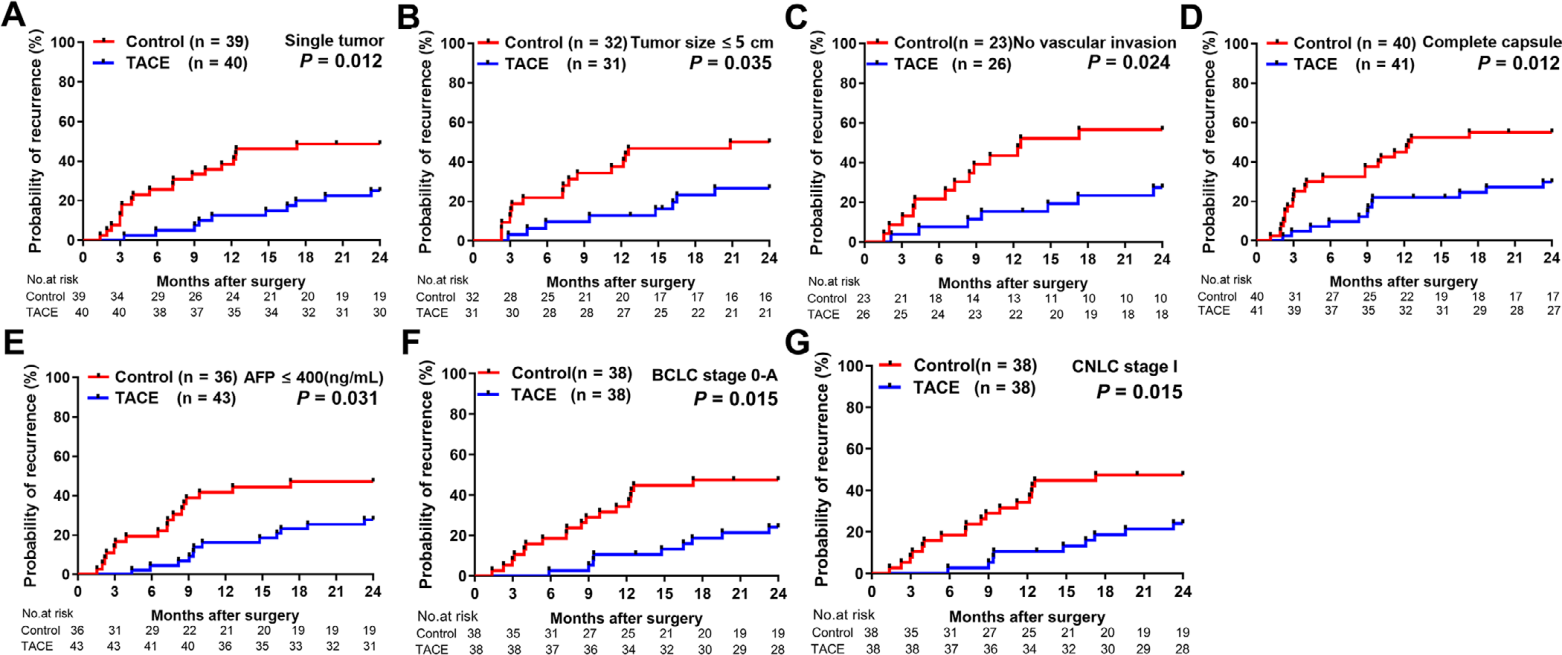
Kaplan‐Meier analysis of early recurrence rates in subgroups of CTC‐positive HCC patients after PSM. Recurrence rates in CTC‐positive patients in the following subgroups: (A) single tumor, (B) tumor diameter <5 cm, (C) no vascular invasion, (D) complete capsule, (E) alpha‐fetoprotein (AFP) level ≤400 ng/mL, (F) Barcelona Clinic Liver Cancer (BCLC) stage 0‐A, or (G) Liver Cancer Guidelines in China (CNLC) stage I

**FIGURE 6:**
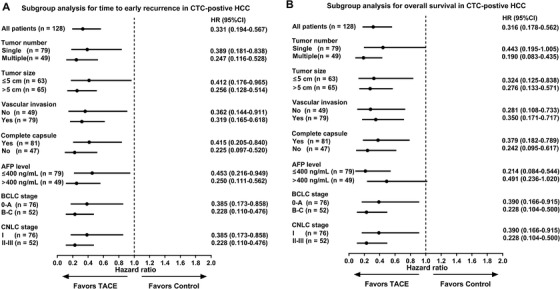
Subgroups analyses for time to early recurrence (A), and OS (B) by Cox regression model based on independent assessment in CTC‐positive HCC patients after PSM **Abbreviations**: AFP, alpha fetoprotein; BCLC, Barcelona Clinic Liver Cancer; CNLC, Liver Cancer Guidelines in China; CT, computed tomography; CTC, circulating tumor cell; DSA, digital subtraction angiography; HCC, hepatocellular carcinoma; OS, overall survival; PSM, propensity score matching; TACE, transcatheter arterial chemoembolization.

Next, the predictive value of positive CTC status for therapeutic benefit of adjuvant TACE was evaluated within clinical subgroups in post‐PSM analysis. Adjuvant TACE significantly reduced early recurrence rates, compared to no TACE treatment, for CTC‐positive patients in the following subgroups: single tumor (25.0% vs 48.7%, *P *= .012), tumor diameter <5 cm (26.7% vs 50.0%, *P *= .035), absence of vascular invasion (27.5% vs 56.5%, *P *= .024), complete tumor capsule (29.8% vs 55.0%, *P *= .012), AFP level ≤400 ng/mL (27.9% vs 47.2%, *P *= .031), BCLC stage 0‐A (24.1% vs 47.4%, *P *= .015), and CNLC stage I (24.1% vs 47.4%, *P *= .015) in TTR at 2 years. Kaplan‐Meier survival analyses for clinical subgroups are shown in Figure 5A‐G. The efficacy of adjuvant TACE on time to early recurrence and OS in exploratory subgroups of CTC‐positive HCC patients was further evaluated using multiple individual Cox models. The most HRs of these analyses for early recurrence and OS were less than 1, indicating that adjuvant TACE is beneficial to most of the subgroups (Figures 6A and 6B).

### 3.5 Toxicity of adjuvant TACE

The adverse events associated with adjuvant TACE were presented in Table S2. Generally, postoperative adjuvant TACE was well tolerated by HCC patients. In the present study, there was no serious complications or adverse events observed in any patient who received adjuvant TACE. The most common adverse events associated with the TACE treatment were nausea or vomiting (43.4%), transient fever (34.9%), and transient deterioration of liver function (ie, elevated levels of ALT, aspartate aminotransferase, bilirubin, and GGT and decreased albumin), which are also common adverse events after adjuvant TACE.

## 4 DISCUSSION

Postoperative tumor recurrence limited the survival of HCC patients after surgery.^1^, ^3^ Postoperative adjuvant TACE is a commonly used adjuvant therapy in preventing tumor recurrence after surgery^17^; however, only certain patients may benefit from adjuvant TACE.^6^, ^18^ CTC detection can be a reliable and versatile means for early cancer detection, prediction of recurrence or metastasis risk, and assessment of response to treatment.^19^ In this retrospective, nonrandomized intervention study, we investigated that whether preoperative CTC status could indicate the treatment benefit of postoperative adjuvant TACE, and PSM was applied to minimize selection bias and confounding factors. Overall, our data indicate that adjuvant TACE can effectively reduce postoperative tumor recurrence, thereby improving OS, in CTC‐positive HCC patients, but the benefits of adjuvant TACE were not observed in CTC‐negative patients in the present study. Specifically, the effect of adjuvant TACE on OS in CTC‐positive patients was achieved primarily via a reduction in the rate of early recurrence (within 2 years), as there was no marked effect on the rate of late recurrence. For CTC‐negative patients, adjuvant TACE did not decrease the early recurrence rate in comparison to untreated patients, likely due to the intrinsic low risk of recurrence in CTC‐negative patients. Thus, preoperative CTC status might be an indicator for evaluating the therapeutic benefit of adjuvant TACE in HCC patients after surgery.

CTCs are easily detected using simple blood draws with minimal risk and therefore could potentially be applicable in clinical management of cancer. In the present study, exploratory subgroup analyses showed that adjuvant TACE can effectively reduce early recurrence rates and provide survival benefit in subgroups of HCC patients that would generally be assessed to have low risk of recurrence and should not be recommended for adjuvant TACE based on traditional clinical indicators (Figures 5 and 6). We consider these results in traditional low‐risk subgroups were not conflict with previous studies, because we chose a novel biomarker to stratify the risk of recurrence, that adjuvant TACE was beneficial to the CTC‐indicated high recurrence risk patients. Therefore, CTC status may be a complementary biomarker of traditional clinical indicators of responsiveness to adjuvant TACE in HCC patients. In addition, although an active postoperative treatment is of great importance, preserving postoperative liver function and avoid excessive treatment is important for HCC patients.^20^ Additional postoperative treatment may be not necessarily needed by CTC‐negative patients. Thus, preoperative CTC status may inform on the optimal use of adjuvant TACE to improve postoperative HCC outcome. A further prospective, multi‐center, large population study is warranted to fully reveal and validate the value of CTC as a biomarker in guiding postoperative adjuvant treatment.

In this study, it was necessary to apply PSM to account for differences in risk factors and clinical features between the TACE and control groups that would otherwise confound the true effect of TACE. Although PSM did not affect conclusions regarding the efficacy of TACE in CTC‐negative patients, PSM was necessary to fully reveal the effects of adjuvant TACE in CTC‐positive patients. Specifically, the clinical outcome of CTC‐positive patients without adjuvant TACE was indicated to be much worse after PSM than it before PSM, and therefore it revealed the benefit of adjuvant TACE in CTC‐positive patients. Given the discrepancies that the patients with adjuvant TACE were more likely to have advanced HCC (Tables 2 and S1) and a higher recurrence risk based on clinical indexes, these results demonstrate the rationality of our matching strategy.

Early recurrence in HCC patients is typically resulted from occult micrometastases, which poses a challenge for early detection and intervention in clinical practice.^4^, ^5^ Adjuvant TACE offers an opportunity for the detection and intervention of these micrometastases in the short‐term after surgery. Accumulating evidences demonstrated that presence of CTCs in circulatory system could decipher the aggressive biological properties of tumor, and identify the patients at high risk of tumor spread.^9^, ^21^, ^22^, ^23^ Our results indicated that the therapeutic effect of TACE treatment in CTC‐positive HCC patients is primarily relied on a reduction of early postoperative recurrence, which may be caused by clinically undetected MRD or occult lesions.^7^, ^24^ The injected lipiodol component of TACE can accumulate selectively in micrometastatic HCC nodules, embolizing the nutrient vessels and delivering high local concentrations of chemotherapy drugs to the micrometastases, and thereby eliminate or suppress the progression of tumor cells.^25^, ^26^ In this study, 15 of 106 (14.2%) patients in TACE group had suspected tumor stains during TACE treatment. Figure S2 is showing typical images demonstrating the adjuvant TACE procedures and effects from a patient who had a suspected target tumor stain in the remnant liver. Among them, 12 patients were CTC‐positive and three patients were CTC‐negative. For the 12 CTC‐positive patients, we found that three patients (25.0%) suffered tumor recurrence within 2 years after adjuvant TACE in the follow‐up period, whereas the 2‐year recurrence rate in our CTC‐positive patients without adjuvant TACE group was 50.1%. These cases also indicated the function of adjuvant TACE in discovering and reducing tumor early recurrence in HCC patients with positive CTCs. Therefore, preoperative CTC evaluation might enable earlier therapeutic intervention like adjuvant TACE at a time when the micrometastatic tumor lesions remain potentially curable.

Our study has limitations. The study is a retrospective study from a single medical center. The value of postoperative CTC in guiding postoperative adjuvant TACE was not evaluated in this study. Second, although PSM analyses were used to enhance intergroup comparisons, unidentified confounders or bias might still influence the results. Third, sample size was a limitation, particularly for subgroup analyses. Finally, the CellSearch system has inherent shortcomings and might not fully evaluate the CTC status of patients.^27^ Going forward, the effectiveness of adjuvant TACE in CTC‐positive HCC patients needs to be validated by a prospective, multi‐center, randomized clinical study.

This study provides new evidence that preoperative CTC status could be a useful indicator for predicting the efficacy of adjuvant TACE in HCC. Adjuvant TACE benefits CTC‐positive HCC patients by reducing early recurrence or delaying the rapid progression of postoperative MRD. Thus, preoperative CTC status is a potentially promising biomarker for the stratification of HCC patients and a clinical indicator for the administration of postoperative adjuvant therapy.

## CONFLICT OF INTEREST

The authors declare that they have no conflict of interest.

## Supporting information


**Supplementary Figure 1. Comparison of recurrence rates and OS between TACE and control groups for all enrolled HCC patients**. Recurrence rates (A) and OS (B) for patients in TACE and control groups.

**Supplementary Figure 2. Typical DSA and CT images demonstrating the adjuvant TACE procedures and effects**. (A) DSA manifestation of a suspected target tumor stain in the remnant liver from a patient with HCC after surgery. (B) DSA manifestation of the effect of adjuvant TACE from the patient. (C) CT manifestation at one month after the adjuvant TACE from the patient.

SUPPORTING INFORMATION

SUPPORTING INFORMATION

## Data Availability

The data that support the findings of this study are available from the corresponding author upon reasonable request.
