# Supplementary figures and images for: Circulating tumor cells are an indicator for the administration of adjuvant transarterial chemoembolization in hepatocellular carcinoma: A single‐center, retrospective, propensity‐matched study
Source: Clin Transl Med. 2020 Jul 23;10(3):e137. doi: 10.1002/ctm2.137 (PMC7418815; doi:10.1002/ctm2.137)

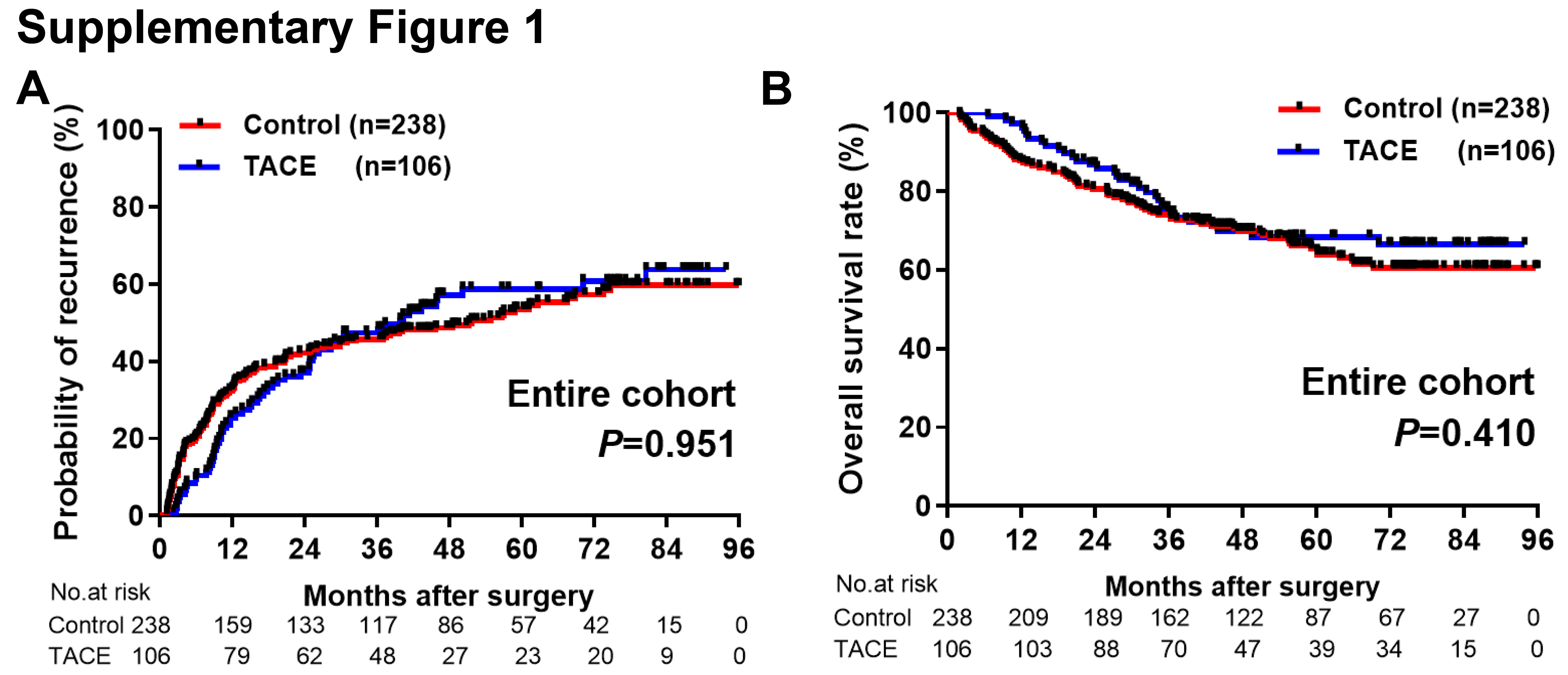

Supplement: Supplementary file 1 — Supplementary Figure 1. Comparison of recurrence rates and OS between TACE and control groups for all enrolled HCC patients. Recurrence rates (A) and OS (B) for patients in TACE and control groups. [file CTM2-10-e137-s001.tif]

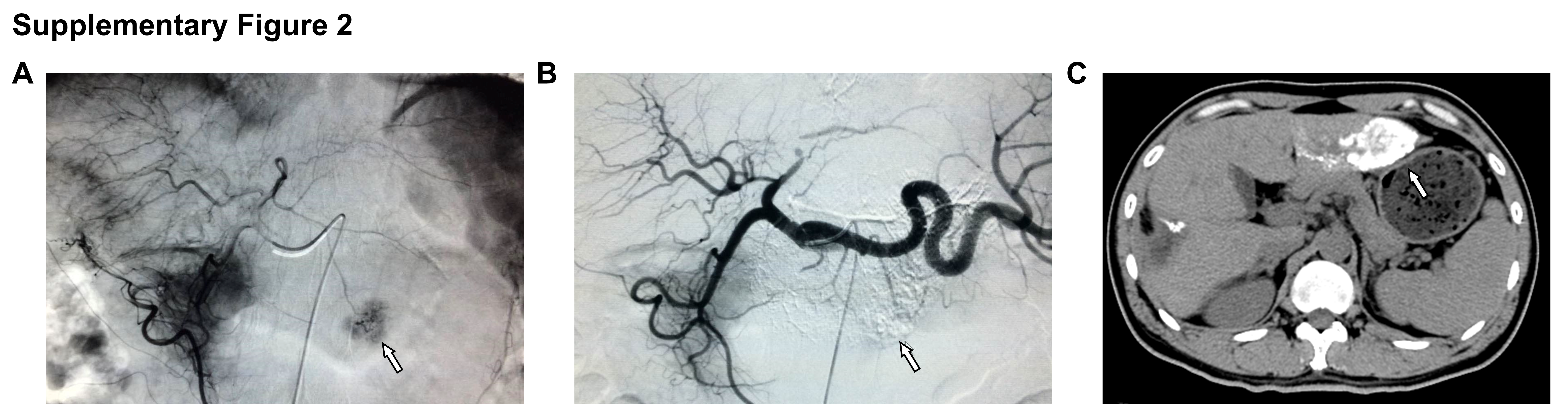

Supplement: Supplementary file 2 — Supplementary Figure 2. Typical DSA and CT images demonstrating the adjuvant TACE procedures and effects. (A) DSA manifestation of a suspected target tumor stain in the remnant liver from a patient with HCC after surgery. (B) DSA manifestation of the effect of adjuvant TACE from the patient. (C) CT manifestation at one month after the adjuvant TACE from the patient. [file CTM2-10-e137-s004.tif]
