# Supplementary material for: Circulating tumor cells are an indicator for the administration of adjuvant transarterial chemoembolization in hepatocellular carcinoma: A single‐center, retrospective, propensity‐matched study
Source: Clin Transl Med. 2020 Jul 23;10(3):e137. doi: 10.1002/ctm2.137 (PMC7418815; doi:10.1002/ctm2.137)
Supplement: Supplementary file 3 — SUPPORTING INFORMATION [file CTM2-10-e137-s002.docx]

**Table S1.** Baseline characteristics of CTC-negative HCC patients.

| Variable |  | | Before propensity matching (n=123) | | |  | After propensity matching  (n=42) | | |
| --- | --- | --- | --- | --- | --- | --- | --- | --- | --- |
|  | | | **Control**  **(n=92**) | **TACE**  **(n=31)** | ***P*** |  | **Control**  **(n=21)** | **TACE**  **(n=21)** | ***P*** |
| Gender | | Male | 81 (88.0%) | 29 (93.5%) | 0.600^a^ |  | 20 (95.2%) | 20 (95.2%) | 1.000^a^ |
|  | | Female | 11 (12.0%) | 2 (6.5%) |  |  | 1 (4.8%) | 1 (4.8%) |  |
| Age (years) | | ≤50 | 31 (33.7%) | 15 (48.4%) | 0.144 |  | 10 (47.6%) | 10 (47.6%) | 1.000 |
|  | | >50 | 61 (66.3%) | 16 (51.6%) |  |  | 11 (52.4%) | 11 (52.4%) |  |
| Tumor number | | Single | 82 (89.1%) | 18 (58.1%) | **<0.001** |  | 14 (66.7%) | 14 (66.7%) | 1.000 |
|  | | Multiple | 10 (10.9%) | 13 (41.9%) |  |  | 7 (33.3%) | 7 (33.3%) |  |
| Tumor diameter | | ≤5 | 70 (76.1%) | 21 (67.7%) | 0.360 |  | 17 (81.0%) | 15 (71.4%) | 0.469 |
| (cm) | | >5 | 22 (23.9%) | 10 (32.3%) |  |  | 4 (19.0%) | 6 (28.6%) |  |
| Tumor capsule | | Complete | 59 (64.1%) | 9 (29.0%) | **0.001** |  | 9 (42.9%) | 8 (38.1%) | 0.753 |
|  | | None | 33 (35.9%) | 22 (71.0%) |  |  | 12 (57.1%) | 13 (61.9%) |  |
| Vascular invasion | | No | 67 (72.8%) | 15 (48.4%) | **0.013** |  | 13 (61.9%) | 13 (61.9%) | 1.000 |
|  | | Yes | 25 (27.2%) | 16 (51.6%) |  |  | 8 (38.1%) | 8 (38.1%) |  |
| Edmondson stage | | Ⅰ-Ⅱ | 66 (71.7%) | 11 (35.5%) | **<0.001** |  | 9 (42.9%) | 11 (52.4%) | 0.537 |
|  | | Ⅲ-Ⅳ | 26 (28.3%) | 20 (64.5%) |  |  | 12 (57.1%) | 10 (47.6%) |  |
| Liver cirrhosis | | No | 47 (51.1%) | 12 (38.7%) | 0.233 |  | 7 (33.3%) | 7 (33.3%) | 1.000 |
|  | | Yes | 45 (48.9%) | 19 (61.3%) |  |  | 14 (66.7%) | 14 (66.7%) |  |
| HBsAg | | Negative | 14 (15.2%) | 6 (19.4%) | 0.589 |  | 5 (23.8%) | 5 (23.8%) | 1.000 |
|  | | Positive | 78 (84.8%) | 25 (80.6%) |  |  | 16 (76.2%) | 16 (76.2%) |  |
| AFP (ng/mL) | | ≤400 | 69 (75.0%) | 23 (74.2%) | 0.929 |  | 16 (76.2%) | 16 (76.2%) | 1.000 |
|  | | >400 | 23 (25.0%) | 8 (25.8%) |  |  | 5 (23.8%) | 5 (23.8%) |  |
| ALT (U/L) | | ≤50 | 79 (85.9%) | 24 (77.4%) | 0.270 |  | 17 (81.0%) | 18 (85.7%) | 1.000^a^ |
|  | | >50 | 13 (14.1%) | 7 (22.6%) |  |  | 4 (19.0%) | 3 (14.3%) |  |
| GGT(U/L) | | ≤60 | 58 (63.0%) | 15 (48.4%) | 0.151 |  | 13 (61.9%) | 11 (52.4%) | 0.533 |
|  | | >60 | 34 (37.0%) | 16 (51.6%) |  |  | 8 (38.1%) | 10 (47.6%) |  |
| Child-Pugh class | | A | 91 (98.9%) | 30 (96.8%) | 0.442^b^ |  | 21(100.0%) | 21 (100.0%) | 1.000 |
|  | | B | 1 (1.1%) | 1 (3.2%) |  |  | 0 | 0 |  |
| BCLC stage | | 0-A | 84 (91.3%) | 17 (54.8%) | **<0.001** |  | 16 (76.2%) | 14 (66.7%) | 0.495 |
|  | | B-C | 8 (8.7%) | 14 (45.2%) |  |  | 5 (23.8%) | 7 (33.3%) |  |
| CNLC stage | | Ⅰ | 84 (91.3%) | 17 (54.8%) | **<0.001** |  | 16 (76.2%) | 14 (66.7%) | 0.495 |
|  | | Ⅱ-Ⅲ | 8 (8.7%) | 14 (45.2%) |  |  | 5 (23.8%) | 7 (33.3%) |  |

^a^ Continuous correction.

^b^ Fisher’s exact test.

Abbreviations: CTC, circulating tumor cell; HCC, hepatocellular carcinoma; TACE, transcatheter arterial chemoembolization; HBsAg, Hepatitis B surface antigen; AFP, alpha-fetoprotein; ALT, alanine aminotransferase; GGT, gamma-glutamyl transpeptidase; BCLC, Barcelona Clinic Liver Cancer staging system; CNLC, Liver Cancer Guidelines in China.
