# Supplementary material for: Circulating tumor cells are an indicator for the administration of adjuvant transarterial chemoembolization in hepatocellular carcinoma: A single‐center, retrospective, propensity‐matched study
Source: Clin Transl Med. 2020 Jul 23;10(3):e137. doi: 10.1002/ctm2.137 (PMC7418815; doi:10.1002/ctm2.137)
Supplement: Supplementary file 4 — SUPPORTING INFORMATION [file CTM2-10-e137-s003.docx]

**Table S2.** Adverse Events for the adjuvant TACE for patients with HCC.

| **Adverse events after adjuvant TACE** | **No. of events (% of patient)** |
| --- | --- |
| **Nausea and/or vomiting** | 46 (43.4%) |
| **Pain** | 29 (27.4%) |
| **Fever** | 37 (34.9%) |
| **Fatigue** | 20 (18.9%) |
| **Increase in ALT/AST** | 30 (28.3%) |
| **Increase in GGT** | 17 (16.0%) |
| **Decrease in albumin** | 8 (7.5%) |
| **Increase in bilirubin** | 13 (12.3%) |

Abbreviations: TACE, transcatheter arterial chemoembolization; HCC, hepatocellular carcinoma; ALT, alanine aminotransferase; AST, aspartate aminotransferase; GGT, gamma-glutamyl transpeptidase.
